# Effect of Sex and Breed on HSPA1A, Blood Stress Indicators and Meat Quality of Lambs

**DOI:** 10.3390/ani10091514

**Published:** 2020-08-27

**Authors:** Thuthuzelwa Stempa, Graeme Bradley

**Affiliations:** Department of Biochemistry and Microbiology, Faculty of Science and Agriculture, University of Fort Hare, Alice 5700, South Africa; gbradley@ufh.ac.za

**Keywords:** adenosine triphosphate, Dorper, heat shock proteins, hypothalamic pituitary axis, lairage, Merino

## Abstract

**Simple Summary:**

Livestock rearing and slaughter methods have received much criticism from various sub-divisions of the society over the years. The root of this criticism is attributed to the growing demand for meat produced from humanely handled animals and issues associated with meat quality and traceability. Ethical arguments around the slaughtering of animals has had less impact on the demand of animal products, however public concern about the welfare of slaughter animals and pre-slaughter stress have been the topical issues. Research on the impact of pre-slaughter stress on animal welfare and meat quality, mostly in pigs and cattle has been well documented. However, there is still paucity of information on the biomarkers of stress in lambs in response to pre-slaughter and its overall impact on meat quality. Therefore, the objective of this study was to examine sex and breed effects on heat shock proteins, blood stress indicators and meat quality attributes from lambs slaughtered at commercial abattoir.

**Abstract:**

The objective of this study was to examine sex and breed effects on heat shock protein 70 (HSPA1A), blood stress indicators and meat quality attributes of lambs. A hundred male and female lambs from the Dorper (n = 50) and Merino (n = 50) breeds were used in this study. Breed and sex had a significant (*p* < 0.05) effect on the levels of plasma HSPA1A and lactate; where the Merino lambs had higher levels than Dorper. The female lambs had higher levels of plasma HSPA1A than male lambs. Significant sex and breed interactions (*p* < 0.05) on the levels of plasma HSPA1A were seen. Females had higher (*p* < 0.05) pHu than males. Dorper lambs had higher (*p* < 0.05) pH_45_, meat lightness, thawing loss and tougher meat the Merino breed. Significant correlations were found amongst plasma stress indicators and meat quality attributes. The results indicate that female lambs were more stressed by the pre-slaughter period than males, while the Merino had a higher physiological stress response compared to the Dorper. However, the Dorper breed produced tougher meat.

## 1. Introduction

Lambs are exposed to a variety of stressors during the pre-slaughter period, such as social mixing, loading and unloading and physical discomfort during the journey [1].Exposure to pre-slaughter stress results in cellular damage hence there is a rapid decline in the intracellular pH [2]. Stressed animals have a higher meat pH and increased heat shock protein (HSP) expression. When animals are stressed the cells produce heat shock protein 70 (HSPA1A) in an attempt to reverse the effects of stress and maintain protein homeostasis [3]. Heat shock proteins are grouped into five classes according to their molecular masses namely, those ranging from 12 to 43 kDa, 60 kDa, 70 kDa, 90 kDa, 100 kDa and they are termed small HSP, HSP60, HSP70, HSP90, HSP100, respectively [4].

High levels of HSP70 (HSPA1A) are very important in the meat industry due to the detrimental effects they can have on the overall quality of meat, due to the negative relationship between HSP70 (HSPA1A) and beef ultimate pH (pHu) [5]. Research on the relationships between HSPs and beef and pork quality have been reported, however there is limited research on the HSP70 and lamb meat quality. Furthermore, ref. [6] reported that HSP27 was negatively correlated with beef tenderness, juiciness and color attributes (L* and a*). However, HSP27 is positively correlated in tenderness pork meat [7].

Exposure to pre-slaughter stress leads to an increase in some stress indicators in the blood such as glucose, lactate, cortisol [8]. Cortisol is a well-known indicator of animal welfare at slaughter [9], which is elevated above basal levels during adverse situations at pre-slaughter [10,11]. Exposure to stress activates the Hypothalamic-Pituitary axis (HPA) and results in the release of catecholamines and glucocorticoids thus activating liver glycogenolysis and elevating the levels of blood glucose [9,12]. Furthermore, increased plasma lactate levels are usually associated with rapid anaerobic glycolysis [13] due to pre-slaughter stress shortly before or during the slaughter process [14].

How in which an animal responds to pre-slaughter stress differs depending on animal-related factors such as individual susceptibility, species, age, sex and breed [15]. Furthermore, meat quality is also affected by animal-related factors such as age, sex and breed [16]. Many researchers have evaluated the relationships between stress indicators such as glucose, lactate, cortisol, creatine kinase, lactate dehydrogenase on the quality of beef and pork from cattle and pigs exposed to pre-slaughter stress [8,17,18,19,20]. However, there is limited information on the effects of sex, breed, pre-slaughter environment on HSPs and their relationship with blood stress indicators and meat quality attributes, especially in lambs. Hence, this study seeks to investigate the sex and breed effects on the levels of HSPA1A, blood stress indicators and meat quality attributes from lambs.

## 2. Materials and Methods

The study was conducted at a high-throughput commercial abattoir in the Buffalo City local municipality of the Eastern Cape Province, South Africa. The abattoir is classified as a high-throughput (A-grade) commercial abattoir because of the large lairage area, abattoir operations and large storage facility [21]. Permission to conduct the study was approved by the Research Ethics Committee of the University of Fort Hare, South Africa (UFH/UREC, MUC371SSTE01).

### 2.1. Animal Management and Slaughter Procedure

A total of 100 eight-month-old male and female lambs consisting of Dorper (n = 50) and Merino (n = 50) breeds were slaughtered at a commercial abattoir. Both the Dorper and Merino breeds consisted of intact rams and non-pregnant females. The lambs were reared at commercial farm mainly for meat production. The lambs were transported from the same farm to the abattoir for a distance of 280 km. On arrival at the abattoir the lambs were placed at the lairages for 12 h. At lairage, the lambs had *ad-libitum* access to water, although feed was not provided to avoid carcass contamination at slaughter. The slaughter procedure was in accordance with the rules and regulations stipulated in the Meat Safety Act No. 4 (2000) where “killing of an animal and other accompanying acts in connection therewith in order to obtain meat and animal products therefrom” [21]. Lambs were electrically stunned at 650 volts for 5 s with concave tongs to induce unconsciousness. After stunning, the throat was cut open with a sharp knife to initiate exsanguination and lambs were hung by the hind legs on the rails to facilitate the bleeding process.

### 2.2. Blood Sample Collection and Storage

Blood samples were collected from each animal within 10 s of exsanguination after stunning and sticking. The blood samples for the analysis of glucose and lactate levels were collected using 10.0 mL disposable Becton Dickinson vacutainer tubes treated with fluoride oxalate (grey top) whereas those for determination of cortisol and heat shock protein 70 (HSPA1A) levels were collected using plasma separating Becton Dickinson vacutainer tubes, Oakville, ON, Canada (SSTTMII, gold top). The blood tubes were centrifuged (Model 5403 Centrifuge, Gatenbay Eppendorf GmbH, Engelsdorp, Germany) at 21 °C for 1000 g for 15 min. The plasma was then placed in 1.5 mL sterile Eppendorf tubes and stored at −80 °C until analysis to avoid loss of bioactivity.

### 2.3. Heat Shock Protein Analysis 70 kDa (HSPA1A)

The analysis of HSPA1A was carried out using the quantitative sandwich Enzyme-Linked Immunosorbent Assay kit (MyBioSource, Cat No. MBS034426, San Diego, CA, USA). The sensitivity of the kit was 0.1 ng/mL. All reagents and samples were brought to room temperature, 30 min before starting the assay procedure. The standards, samples and control were added in duplicate on the micro-ELISA striplate. HPR-conjugate reagent (100 µL) was added to each well and the plate was covered with a closure plate membrane and incubated for 60 min at 37 °C. The plate was then washed four times manually using the wash solution. After washing, 50 µL of chromogen solution A and 50 µL of chromogen solution B were added to each well successively. The plate was protected from light and incubated for 15 min at 37 °C. After incubation 50 µL of Stop solution was added to each well and the color changed from blue to yellow. The optical density (O.D.) was read at 450 nm using an ELISA reader (SynergyMx BioTek, model SN 236255, Winooski, VT, USA). The duplicate readings were averaged for each standard and samples to subtract average optical density of the blank/control.

### 2.4. Plasma Cortisol Determination

Plasma cortisol analysis was carried out using a quantitative sandwich ELISA kit for the detection of sheep cortisol in serum (Bioassay technology laboratory, Cat. No. E0003Sh, Shanghai, China). The sensitivity of the kit was 0.34 ng/mL. All reagents and samples were brought to room temperature 30 min before starting the assay procedure. The standards (50 µL) were added to the standard wells. Samples (40 µL) were added into to sample wells and 10 µL anti-cortisol antibody was added to the sample wells. Then streptavidin-HRP (50 µL) was added to sample and standard wells but not in the blank well. After this, the plate was covered with a plate sealer and incubated for 60 min at 37 °C. The sealer was removed and the plate was washed five times using manual washing with buffer. Substrate A solution (50 µL) was added to each well and then 50 µL of substrate B solution was added to each well. The plate was covered and incubated for 10 min in the dark at 37 °C. The stop solution was then added to each well and the color changed from blue to yellow immediately. The optical density (O.D.) was read at 450 nm using an ELISA reader (SynergyMx BioTek, model SN 236255, Winooski, USA).

### 2.5. Glucose and Lactate Determination

Laboratory analysis of plasma glucose and lactate concentrations was carried out using an Olympus AU400 automated chemistry analyzer (Olympus Optical Co. Ltd., Melville, NY, USA) and the Olympus reagent kit for glucose and lactate (Olympus Cat. No. OSR6193).

### 2.6. Carcass Description

After slaughter, the carcasses were weighed to determine the hot carcass weight (HCW). The carcasses were then chilled at a mean temperature of 3–4 °C. After chilling the carcasses were weighed again to attain the cold carcass weight (CCW). Carcass fatness (CF) in millimeters (mm) and conformation scores (1 very flat–5 very round) were determined using visual appraisal according to the methods for red meat classification in South Africa. All the lambs used in this study had a conformation score of 3 (medium) and a carcass fatness score of 2 (1 £ CF £ 3 mm) as shown in Table 1 [22].

### 2.7. pH and Temperature Measurements

Carcass pH and temperature were measured on the right side of each carcass by inserting the piercing probe in the longissimus muscle between the 12th and 13th ribs at 45 min (pH_45min_, T_m45min_) and at 24 h (pHu, T_m24h_) after slaughter using a portable pH meter (Hach Lange GMBH HQ11d, Düsseldorf, Germany ) which was calibrated in buffers with pH 4.00 and 7.00.

### 2.8. Meat Quality Measurements

A hundred-meat samples (~250 g & 100 mm thick) were harvested from *the longissimus thoracis et lumborum* (LTL) muscle from the left side of each carcass between the 10th and 12th rib in the direction of the rump for meat quality measurements. The samples were vacuum packed and transported to the laboratory in a cooler box half filled with ice packs.

Meat samples were further processed from 100 mm thick into 20 mm steaks using a band saw for Commission International De L‘Éclairage Laboratory [23] color measurements at 24 h post-mortem. The sample’s surface was exposed to air for 30 min to facilitate ‘blooming’ before measurements were taken. Color variables [lightness (L*), redness (a*), yellowness (b*)] were measured using a BYK-Gardner 6692 Color-guide 45/0 glass sealed, with a 20 mm diameter measurement area and illuminant D65-daylight, 10° observation angle. The psychometric hue angle (H*) was calculated as—Hue angle = [tan−1 (b*) / (a*)]; and the psychometric chroma (C*) was calculated as—Chroma = (a^2^ + b^2^) ^0.5^ [24].

### 2.9. Thawing Loss, Cooking Loss and Warner Bratzler Shear Force

After pH and color determination at 24 h post-mortem, the samples were vacuum packed and placed at −20°C refrigerator for seven days. At day seven the frozen samples were weighed (AE Adam Nimbus Precision Balance NBL 214i, Shanghai, China) and allowed to thaw for 10 h at room temperature of 20–22 °C, after thawing the samples were reweighed. The recorded weight differences were expressed as thawing loss % (TL%).

The samples were then placed in polyethene plastic bags and cooked in a water bath pre-heated to 72 °C for 45 min [25]. After cooking, meat samples were cooled to room temperature (±20 °C). The samples were then re-weighed to calculate the amount of water lost during cooking. The results were expressed as the cooking loss percentages (CL%).

Following the thawing and cooking loss measurements, Warner-Bratzler Shear Force (WBSF) test was done. From each sample, three subsamples of approximately 12.7 mm core diameter were extracted parallel to the long axis of the muscle fibers [25]. Each core was sheared once through the center at an angle perpendicular to the direction of the fiber using the Warner-Bratzler shear device attached to the Universal Instron apparatus (Model 3344, crosshead speed = 400 mm/min, Norwood, MA, USA). The WBSF was measured as the peak force (Newtons) average for three cores per sample.

### 2.10. Statistical Analysis

The effect of sex and breed on plasma HSPA1A, cortisol, glucose, lactate HCW, CCM, CF, meat pH, meat temperature, L*, a*, b*, C* and H* from Dorper and Merino lambs were analyzed using the PROC GLM procedure of SAS (2009). Significant differences between least square group means were compared using PDIFF test pf SAS (2009). The following statistical model was used—Y_ijk_ = μ + α_i_ + β_j_ + (αβ)_ij_ + ε_ijk_ where Y_ijk_ = response variables (HSPA1A, cortisol, glucose, lactate, HCW, CCW, CF, pH_45min_, pH_24h_, T_m45min_, T_m24h_, L*, a*, b*, C*, H* and WBSF); μ = overall mean; α_i_ = ith effect of sex (female and male); β_j_ = jth effect of breed (Dorper and Merino); (αβ)_ij_ = interaction between sex and breed; ε_ijk_ = Random error.

The strength of relationships amongst plasma HSPA1A, cortisol, glucose, lactate, HCW, CCW, CF, pH_45min_, pH_24h_, T_m45min_, T_m24h_, L*, a*, b*, C*, H* and WBSF were determined using Pearson’s correlation coefficient [26]. Significant differences between the means were tested using Least Significant Differences (LSD) at *p* < 0.05.

## 3. Results

### 3.1. Sex and Breed Effects on Blood Stress Indicators

Results for the effects of sex and breed on plasma HSPA1A, cortisol, glucose and lactate from Dorper and Merino lambs with sex breed interaction are shown in Table 2. Differences were observed for the breed (*p* < 0.01) and sex (*p* < 0.05) effects on the levels of plasma HSPA1A. Significant sex and breed interactions were observed on plasma HSPA1A levels. The Merino breed had higher levels of plasma HSPA1A than the Dorper. Female lambs had higher levels of plasma HSPA1A compared to the male lambs. Furthermore, breed had a significant (*p* < 0.001) effect on the levels of plasma lactate, the Merino breeds had higher levels of lactate compared to the Dorper.

### 3.2. Sex and Breed Effects on Carcass and Meat Quality Attributes

Results for the effects of sex and breed on carcass and meat quality from the LTL of Dorper and Merino lambs with sex*breed interaction are shown in Table 3. Breed had a significant effect (*p* < 0.001) on the cold carcass weight (CCW) and hot carcass weight (HCW) where the Merino had a higher CCW and HCW compared to the Dorper breed. Meat pH_45min_ was significantly (*p* < 0.05) affected by breed, where the Dorper had a higher pH value compared to the Merino breed. Meat Tm45min was also affected (*p* < 0.001) by breed, where the Merino had a higher meat temperature compared to the Dorper breed. Breed had an effect (*p* < 0.001) on the meat Tm 24 h post-mortem, where the Dorper had a higher meat temperature compared to the Merino breed. Meat L* was affected (*p* < 0.001) by breed, where the Dorper had a higher L* color compared to the Merino breed. Thawing loss was significantly (*p* < 0.05) affected by breed; the Dorper had a higher thawing loss compared to the Merino breed. The Dorper breed produced tougher meat compared to the Merino breed. Sex had a significant effect on the pHu, where the female lambs had a higher pHu compared to male lambs. Furthermore, sex and breed interaction was significant on meat cooking loss.

### 3.3. Pearson’s Correlations (r) for Plasma Heat Shock Proteins, Stress Indicators, Carcass and Meat Quality Attributes from Lambs Slaughtered at a Commercial Abattoir

The relationships amongst plasma heat shock proteins, stress indicators, carcass and meat quality attributes from lambs slaughtered at a commercial abattoir are shown in Table 4. A significant positive (*p* < 0.05) correlation was observed between plasma cortisol and meat redness. Furthermore, plasma lactate was seen to have a strong positive (*p* < 0.01) correlation with meat tenderness. Positive relationship (*p* < 0.05) exists amongst plasma HSPA1A and hot carcass mass and carcass fatness. Significant correlations were observed between L*, a* (r = −45) and H* (r = 0.001). Strong positive (*p* < 0.001) correlations were observed between a*, b* (r = 0.46) and C*(r = 0.92). Lastly, strong positive (*p* < 0.001) correlations were observed between a*, C*(r = 0.75) and H* (r = 0.78).

## 4. Discussion

### 4.1. Sex and Breed Effects on Blood Stress Indicators

Breed differences in stress response can be linked to complex genetic factors associated with selection for production traits [27]. Even though, cortisol in this study was not significantly different in lambs in this study, exposure to stress also leads to expression of HSPA1A. Heat shock protein 70 kDa (HSPA1A) consists of preserved stress proteins that are released in response to stressors [28]. Furthermore, the expression of plasma HSPA1A improves intracellular homeostasis since it protects against cell destruction by preventing protein degradation and assisting in the folding of proteins [29]. When lambs are exposed to stressful conditions, as a means to deal with maintaining homeostasis there is an increase in the levels of stress response hormones in the blood [30]. It also believed that selection for production traits is usually linked with temperament [31]. Furthermore, expression of plasma HSPA1A varies depending on individual adaptability to environmental and intracellular changes of pH during exposure to stress [32].

The Merino breed had higher levels of plasma lactate. Animals with higher plasma lactate at slaughter tend to have more flighty behavior at slaughter hence they use up more glycogen reserves thus resulting in meat with a high ultimate pH. Hence, is animals with flighty temperaments during handling tend to produce higher levels of plasma stress indicators [18].

### 4.2. Sex and Breed Effects on Carcass and Meat Quality Attributes

Breed differences in HCW and CCW can be attributed to several factors that influence breed weight differences such as conversion efficiency, physiological responses, selection for production traits [33]. The Dorper breed is a major sheep breed that is normally extensively reared for mainly for meat whereas the Merino is a dual purpose breed [31]. Female lambs produced higher pHu values compared to the male lambs. These results contradict those reported by Johnson et al. [34], who reported rams to have a higher pHu compared to the ewes. The pHu values (>6.00) for females in this study were higher than the acceptable normal pHu range of 5.5–5.8 [35]. This is an indication that, female lambs were more stressed at slaughter. Furthermore, these results indicate that the females were more susceptible and stressed compared to the male lambs during the pre-slaughter period. The female lambs also had depleted glycogen reserves due to glucogenolysis induced by a stress response meaning that insufficient lactate would be produced post-mortem, resulting in a pHu higher than the acceptable range [18]. Furthermore, females might have produced meat with reduced the quality compared to the males, since meat pH usually influences physic-chemical properties (water holding capacity, color and tenderness) [36]. However, Stempa et al. [37] argued that female lambs tend to produce better meat quality compared to male lambs.

Breed differences have an impact on the color of meat and the Merino breed has been reported to have a high haem-pigment which causes the meat to be redder in the muscles compared to other breeds. Furthermore, lamb meat color can be influenced by diet [38]. The lambs in this study were grazing on natural pastures which may have influenced the L* values. Other authors found similar results and they reported that the Merino breed produced darker meat and a less acceptable color [39,40]. These results are contrary to those reported by Cloete et al. [41] who reported higher L* values.

Meat tenderness is an important meat quality attribute in lamb as it has a massive bearing on the lamb eating quality and consumer acceptance [42]. Lamb meat with a WBSF values above 54.93 N are considered tough by consumers [43]. Using this criteria the meat produced by the lambs in this study were relatively tender with WBSF values < 40 Newtons. However, differences in breeds might be lamb meat tenderness is affected by breed differences in connective tissue content [44]. These results are similar to those reported by Pannier et al. [42]. However, other authors’ findings are contrary to the results in this study, where meat from Merino lambs was having higher WBSF values [45,46].

### 4.3. Pearson’s Correlations (r) for Plasma Heat Shock Proteins, Stress Indicators, Carcass and Meat Quality Attributes from Lambs Slaughtered at a Commercial Abattoir

When the glycogen is being broken down lactate accumulates in the blood ante-mortem. When these lambs are slaughtered, the glycogen reserves are insufficient to lower the pH of meat. Thus, meat has a high pHu and has a high WBSF thus tougher meat is produced [47,48]. Exposure to pre-slaughter stress conditions results in the release of plasma cortisol and lactate as physiological stress response, which leads to a rapid depletion of glycogen reserves [49]. Meat pHu reached when the conversion of glycogen to lactate is complete and the value is 5.8 or less is to be considered desirable [35]. Hence, lactate can have an indirect relationship with meat quality attributes such as WBSF due to its relationship with pH. These results contradict those reported by Chulayo et al. [5], who reported a negative correlation between HSPA1A and meat quality attributes in cattle exposed to pre-slaughter stress. Significant correlations on meat color attributes (L*, a*, b*) were observed by Lorentzen and Vangen [50]. CIE (L*, a*, b*, C* and H*) values are used to quantify meat color [51]. Meat color is an important quality attribute which consumer associate with freshness and quality [39]. Meat with a brown color is usually regarded as poor quality compared to meat with a bright red color. The significant relationships between stress indicators cortisol and meat redness can be attributed to the circulating levels of cortisol, which tend to recover less rapidly and slower compared with other stress indicators. High cortisol levels have an impact on the lamb quality, which reflects the pre-slaughter period [52].

## 5. Conclusions

The results indicate that the Merino and the female lambs were more stressed during pre-slaughter period as they produced higher levels of plasma HSPA1A and lactate. Furthermore, female lambs produced meat with high ultimate pH. This is an indication that sex has an effect on the pre-slaughter stress response and reduced the overall meat quality. However, breed only had an effect on pre-slaughter stress response but did not affect the quality of meat. The Dorper breed produced lower quality meat compared to the Merino breed. Whilst the Merino breed had higher levels of plasma HSPA1A. Significant correlations were found amongst plasma stress indicators and meat quality attributes. Exposure to pre-slaughter stress should be reduced in order to improve the welfare of lambs to ensure meat quality is not reduced.

## Figures and Tables

**Table 1:** Animal and carcass description of 8-month-old lambs.

Breed	Sex	Avg Live Weights (kg)	Conformation	Carcass Fatness (mm)
Dorper (n = 50)	Non-pregnant ewes	36.40	3	2
	Intact rams	39.50	3	2
Merino (n = 50)	Non-pregnant ewes	38.00	3	2
	Intact rams	43.00	3	2

Conformation scores 1 = very flat to 5 = very round, fat depth was measured in mm from the back of the carcass.

**Table 2:** LSMeans (± SD) of plasma HSPA1A, cortisol, glucose and lactate of Dorper and Merino lambs and sex*breed interactions.

Parameters	Breed		Sex		Breed*Sex Interaction		Breed*Sex Interaction		*p*-value		
	Dorper (D) (*n* = 50)	Merino (M) (*n* = 50)	Female (F) (*n* = 51)	Male (M) (*n* = 49)	D*M	D*F	M*F	M*M	Breed (B)	Sex (S)	B*S
Plasma HSPA1A (ng/mL)	15.94 ± 4.405	34.89 ± 4.462	32.86 ± 4.610	17.97 ± 4.250	16.15 ± 6.229	15.74 ± 6.229	49.99 ± 6.796	19.79 ± 5.783	0.0032 **	0.0195 *	0.0164 *
Plasma Cortisol (ng/mL)	49.09 ± 2.483	50.64 ± 2.516	50.52 ± 2.599	49.22 ± 2.396	49.59 ± 3.512	48.58 ± 3.512	52.44 ± 3.832	48.84 ± 3.261	0.6618	0.7140	0.5157
Plasma Glucose (mmol/L)	4.41 ± 0.165	4.26 ± 0.167	4.14 ± 0.173	4.53 ± 0.159	4.52 ± 0.233	4.30 ± 0.233	3.99 ± 0.255	4.53 ± 0.217	0.5248	0.1050	0.4857
Plasma Lactate (mmol/L)	3.58 ± 0.232	5.01 ± 0.235	4.29 ± 0.242	4.29 ± 0.224	3.56 ± 0.328	3.61 ± 0.328	4.98 ± 0.358	5.03 ± 0.305	<0.0001 ***	0.9968	0.8879

Significant differences at *p* < 0.05 *, *p* < 0.01 **, *p* < 0.001 ***, HSPA1A—Heat shock protein 70, ng/mL—nanogram/ milliliter, mmol/L—millimole/Liter.

**Table 3:** LSMeans (±SD) of carcass characteristics and *the longissimus thoracis et lumborum* meat quality of Dorper and Merino lambs and sex*breed interactions.

Parameters	Breed		Sex		Breed * Sex Interaction		Breed * Sex Interaction		*p*-Value		
	Dorper (n = 50)	Merino (n = 50)	Female (n = 51)	Male (n = 49)	D*M	D*F	M*F	M*M	Breed (B)	Sex (S)	B*S
HCW (kg)	19.71 ± 0.344	22.96 ± 0.349	21.42 ± 0.360	21.25 ± 0.332	19.51 ± 0.487	19.90 ± 0.487	22.95 ± 0.531	22.97 ± 0.452	<0.0001 ***	0.7231	0.6754
CCW (kg)	18.74 ± 0.409	22.27 ± 0.415	20.76 ± 0.429	20.24 ± 0.395	18.19 ± 0.579	19.30 ± 0.579	22.26 ± 0.632	22.29 ± 0.538	<0.0001 ***	0.3590	0.3337
CF (mm)	2.40 ± 0.151	2.53 ± 0.152	2.56 ± 0.158	2.37 ± 0.145	2.40 ± 0.213	2.40 ± 0.213	2.71 ± 0.232	2.34 ± 0.198	0.5470	0.3909	0.3909
pH_45min_	6.06 ± 0.022	5.99 ± 0.022	6.05 ± 0.022	6.01 ± 0.021	6.05 ± 0.031	6.08 ± 0.031	6.01 ± 0.034	5.98 ± 0.029	0.0307 *	0.2640	0.9618
T_m45min_ (°C)	22.16 ± 0.206	23.29 ± 0.209	22.89 ± 0.215	22.56 ± 0.198	21.94 ± 0.291	22.38 ± 0.291	23.40 ± 0.318	23.18 ± 0.270	0.0002 ***	0.2589	0.7151
pH_u_	5.99 ± 0.021	5.99 ± 0.021	6.03 ± 0.022	5.95 ± 0.020	5.93 ± 0.030	6.05 ± 0.030	6.02 ± 0.032	5.98 ± 0.028	0.8873	0.0087 *	0.1644
T_m24h_ (°C)	25.95 ± 0.073	25.42 ± 0.073	25.74 ± 0.075	25.62 ± 0.070	25.88 ± 0.103	26.02 ± 0.103	25.47 ± 0.112	25.36 ± 0.095	<0.0001 ***	0.2300	0.8823
L*	41.27 ± 0.483	37.33 ± 0.489	39.67 ± 0.505	38.93 ± 0.465	40.28 ± 0.683	42.26 ± 0.683	37.08 ± 0.745	37.58 ± 0.634	<0.0001 ***	0.2865	0.0742
a*	16.73 ± 0.269	16.91 ± 0.272	16.69 ± 0.281	16.94 ± 0.259	17.06 ± 0.380	16.40 ± 0.380	16.98 ± 0.415	16.83 ± 0.353	0.6463	0.5119	0.2887
b*	10.20 ± 0.262	9.92 ± 0.265	10.19 ± 0.273	9.93 ± 0.252	10.062 ± 0.370	10.33 ± 0.370	10.04 ± 0.404	9.799±0.343	0.4615	0.4957	0.9715
C*	19.66 ± 0.301	19.65 ± 0.310	19.62 ± 0.320	19.69 ± 0.295	19.86 ± 0.432	19.47 ± 0.432	19.77 ± 0.472	19.52 ± 0.401	0.9666	0.8677	0.4554
H*	0.55 ± 0.011	0.51 ± 0.011	0.55 ± 0.011	0.53 ± 0.011	0.53 ± 0.016	0.56 ± 0.016	0.53 ± 0.071	0.53 ± 0.015	0.3722	0.2857	0.4968
TL%	14.51 ± 0.808	11.62 ± 0.819	12.53 ± 0.846	13.61 ± 0.780	13.70 ± 1.143	15.33 ± 1.143	9.727 ± 1.25	13.52 ± 1.061	0.0137 *	0.3505	0.0203 *
CL%	26.90 ± 1.271	28.42 ± 1.287	28.49 ± 1.330	26.82 ± 1.226	23.89 ± 1.797	29.91 ± 1.797	27.08 ± 1.961	29.75 ± 1.669	0.4043	0.3375	0.0181 *
WBSF(N)	37.78 ± 1.230	37.52 ± 1.246	34.43 ± 1.287	34.87 ± 1.186	33.30 ± 1.739	30.26 ± 1.739	38.61 ± 1.897	36.43 ± 1.615	0.0015 **	0.8037	0.1387

Significant differences at *p* < 0.05 *, *p* < 0.01 **, *p* < 0.001 ***, HCW—hot carcass weight, CCW—cold carcass weight, CF—carcass fatness, pH_45min_—pH at 45 min, T_m45min_—meat temperature at 45 min, pHu—ultimate pH (pH at 24 h), T_m24h_—meat temperature at 24 h, L*—Lightness, a*—Yellowness, b*—Redness, C*—Chroma, H*—Hue angle, TL%—thawing loss percentage, CL%—cooking loss percentage, WBSF—Warner Braztler shear force, kg—kilogram, %—percentage, N—Newtons, mm—millimeter, °C—degrees Celsius.

**Table 4:** Pearson’s correlations coefficients (r) for blood heat shock proteins, stress indicators, carcass and meat quality attributes from lambs slaughtered at a commercial abattoir.

N = 100	Cort	Lact	Glu	HSPA1A	HCW	CCW	CF	pH_45min_	T_m45min_	pHu	T_m24h_	L*	a*	b*	C*	H*	TL%	CL%	WBSF(N)
Cort	-	0.13 ^ns^	0.09 ^ns^	0.17 ^ns^	−0.08 ^ns^	−0.08 ^ns^	0.11 ^ns^	0.02 ^ns^	−0.23 ^ns^	−0.02 ^ns^	0.06 ^ns^	0.21 ^ns^	−0.34 *	−0.01 ^ns^	−0.31 *	0.16 ^ns^	−0.10 ^ns^	0.05 ^ns^	0.15 ^ns^
Lact		-	0.14 ^ns^	0.08 ^ns^	0.24 ^ns^	0.24 ^ns^	0.03 ^ns^	−0.22 ^ns^	0.24 ^ns^	−0.13 ^ns^	−0.35 *	−0.26 ^ns^	0.06 ^ns^	0.09 ^ns^	0.07 ^ns^	0.07 ^ns^	−0.11 ^ns^	0.05 ^ns^	0.39 **
Glu			-	−0.08 ^ns^	−0.08 ^ns^	0.00 ^ns^	−0.08 ^ns^	−0.02 ^ns^	0.07 ^ns^	−0.08 ^ns^	0.06 ^ns^	0.03 ^ns^	0.01 ^ns^	−0.15 ^ns^	−0.05 ^ns^	−0.15 ^ns^	0.15 ^ns^	0.05 ^ns^	0.09 ^ns^
HSPA1A				-	0.32 *	0.19 ^ns^	0.34 *	0.01 ^ns^	0.24 ^ns^	0.21 ^ns^	0.05 ^ns^	−0.01 ^ns^	0.25 ^ns^	0.37 **	0.34 *	0.21 ^ns^	0.07 ^ns^	0.21 ^ns^	−0.15 ^ns^
HCW					-	0.99 ***	0.35 ***	−0.19 ^ns^	0.09 ^ns^	-0.06 ^ns^	0.04 ^ns^	0.08^ns^	−0.04 ^ns^	−0.03 ^ns^	−0.04 ^ns^	−0.03 ^ns^	-0.11 ^ns^	−0.03 ^ns^	0.07 ^ns^
CCW						-	0.35 ***	−0.06 ^ns^	0.38 **	0.08 ^ns^	−0.04 ^ns^	0.05 ^ns^	−0.02 ^ns^	−0.03 ^ns^	−0.03 ^ns^	−0.04 ^ns^	-0.11 ^ns^	0.04 ^ns^	0.09 ^ns^
CF							-	0.01 ^ns^	−0.08 ^ns^	0.16 ^ns^	0.02 ^ns^	−0.05 ^ns^	0.05 ^ns^	−0.12 ^ns^	0.05 ^ns^	0.03 ^ns^	-0.10 ^ns^	−0.10^ns^	0.56 ***
pH_45min_								-	−0.15 ^ns^	0.11 ^ns^	0.06 ^ns^	−0.08 ^ns^	−0.04 ^ns^	0.05 ^ns^	0.05 ^ns^	0.05 ^ns^	0.04 ^ns^	−0.15 ^ns^	0.12 ^ns^
T_m45min_									-	−0.02 ^ns^	−0.08 ^ns^	0.28 ^ns^	−0.22 ^ns^	0.09 ^ns^	−0.13 ^ns^	0.22 ^ns^	0.18 ^ns^	−0.08 ^ns^	−0.04 ^ns^
pHu										-	0.08 ^ns^	−0.24 ^ns^	0.23 ^ns^	−0.04 ^ns^	0.15 ^ns^	−0.21 ^ns^	0.20 ^ns^	0.24 ^ns^	−0.05 ^ns^
T_m24h_											-	−0.10 ^ns^	0.08 ^ns^	−0.03 ^ns^	0.05 ^ns^	0.07 ^ns^	−0.06 ^ns^	−001 ^ns^	−0.12 ^ns^
L*												-	−0.65 ***	0.10 ^ns^	−0.48 ***	0.14 ^ns^	0.21 **	−0.12 ^ns^	−0.22 ^ns^
a*													-	0.59 *	−0.05 ^ns^	0.16 **	−0.12 ^ns^	0.09^ns^	0.20 *
b*														-	0.84 ***	0.93 ***	0.09 ^ns^	−0.20 *	0.03 ^ns^
C*															-	0.17 ^ns^	0.19 ^ns^	−0.20 *	0.18 ^ns^
H*																-	0.13 ^ns^	0.13 ^ns^	−0.07 ^ns^
TL%																	-	0.13 ^ns^	−0.23 *
CL%																		-	0.00 ^ns^
WBSF (N)																			-

Significant differences at *p* < 0.05 *, *p* < 0.01 **, *p* < 0.001 ***, Glu—plasma glucose, Lact—plasma lactate, Cort—plasma cortisol, HSPA1A—Plasma heat shock protein, HCW—hot carcass weight, CCW—cold carcass weight, CF—carcass fatness, pH_45min_—pH at 45 min, T_m45min_—meat temperature at 45 min, pHu—pH at 24 h, T_m24h_—meat temperature at 24 h, L*—Lightness, a*—Yellowness, b*—Redness, C*—Chroma, H*—Hue angle, TL—thawing loss percentage, CL%—cooking loss percentage and WBSF—Warner Braztler shear force in Newtons.
